# Analysis of the Waggle Dance Motion of Honeybees for the Design of a Biomimetic Honeybee Robot

**DOI:** 10.1371/journal.pone.0021354

**Published:** 2011-08-03

**Authors:** Tim Landgraf, Raúl Rojas, Hai Nguyen, Fabian Kriegel, Katja Stettin

**Affiliations:** 1 Department of Mathematics and Computer Science, Free University Berlin, Berlin, Germany; 2 Department of Zoology, University of Cambridge, Cambridge, United Kingdom; Imperial College London, United Kingdom

## Abstract

The honeybee dance “language” is one of the most popular examples of information transfer in the animal world. Today, more than 60 years after its discovery it still remains unknown how follower bees decode the information contained in the dance. In order to build a robotic honeybee that allows a deeper investigation of the communication process we have recorded hundreds of videos of waggle dances. In this paper we analyze the statistics of visually captured high-precision dance trajectories of European honeybees (*Apis mellifera carnica*). The trajectories were produced using a novel automatic tracking system and represent the most detailed honeybee dance motion information available. Although honeybee dances seem very variable, some properties turned out to be invariant. We use these properties as a minimal set of parameters that enables us to model the honeybee dance motion. We provide a detailed statistical description of various dance properties that have not been characterized before and discuss the role of particular dance components in the commmunication process.

## Introduction

After returning from a valuable food source honeybee foragers move vigorously, in a highly stereotypical pattern, on the comb surface. Intriguingly, an increasing number of nestmates can be observed visiting the feeding site only a few minutes past that event ([1], [2]). Karl von Frisch discovered that the behavior, he called tail-wagging dance, not only informs foragers about the mere existence of a food source. At least to us humans, it even communicates the polar coordinates of a valuable field location ([1]) enabling the colony to direct and coordinate foraging activities. This is a remarkable example of symbolic information transfer in the animal world and has attracted much interest from different scientific domains.

A tail-wagging forager moves on the vertical comb in an approximate figure of eight. In the central part - the so called waggle run - it throws its body from side to side in a pendulum like motion at a frequency of about 13 Hz. Throughout that run the dancer holds tight to the comb moving forward in a rather straight line. Each waggle-phase is followed by a return-phase, in which the dancer circles back to the approximate starting point of the previous waggle run, alternatingly performed clockwise and counter-clockwise. Interestingly, dance parameters reflect feeding site properties. In the waggle phase, the body angle with respect to gravity approximates the direction to the feeder relative to the sun's azimuth. The length and duration of the waggle run correlate highly with the distance to the target location ([1],[2]). In addition to direction and distance, the dance communicates the profitability or quality of the food source with respect to the current hive's needs. Foragers tend to dance more lively and perform longer dances when feeding on a highly profitable source ([3], [2]).

The bees actively pursuing the dancer, commonly called follower bees, are most likely to be recruited after joining several dance periods. In that process they tend to remain in close contact with the dancer and detect a variety of stimuli. Antennal contacts with the body of the dancer are frequently observable and likely transmit information about the dancer's body orientation ([4], [5], [6]). Wing bursts in the waggle run produce laminar air flows, three-dimensional fields of short-ranged air particle oscillations and comb vibrations that might as well deliver meaningful multisensory input at the receiver side of the communication ([7], [8], [9], [10]). An increased thoracic temperature is characteristic to dancers and also encodes food quality ([11]). Recently, a dance-specific scent has been reported ([12]) as yet another possible signal. Floral odors and regurgitated food samples are associated cues. However, which of the many stimuli is actually used for the communication could not yet be clarified.

Moreover, the dance communication involves highly complex cognitive tasks. A dancer bee extracts and translates field site properties from the memory formed on her foraging trips - the dance is no instantaneous or spontaneous response nor a signal for an immediate action. Follower bees “read” the dance, translate their sensory input into the remote target location and find the feeder even with large detours around obstacles like high buildings or hills. The bee dance community has gathered an amazing amount of knowledge on navigation, memory and communication in honeybees ([13]) and we can rely on compelling evidence indicating that honeybees actually evaluate and use the information encoded in the dance. However, after more than 60 years of intense research it is still unknown how exactly information is encoded in the dance and how it is decoded by the followers. Which of the many stimuli carry information? Can we assign specific meanings to single stimuli? How do the followers use that complex mosaic of stimuli they perceive and how are they mapped to their subsequent behavior in the field? Do bees extract such abstract concepts as angles to integrate them in and read them from the dance? Or is the encoding and the decoding process more of a memory playback and recording, respectively?

To tackle a part of these questions we are developing a robotic honeybee that can emulate all known stimuli ([14]). Using a robot we can control every signal individually and observe the effect of arbitrary stimulus combinations. To this end we track the behavior of the bees that had contact to the honeybee robot by highly detailed video recordings in the hive and harmonic radar ([15]) in the field. Through that work we will be able to identify essential communication signals and deduce signal media and sensory modalities.

In order to mimic the dance as realistic as possible we captured the dance motion via an automatic tracking program from video recordings of natural dances. This paper covers the analysis of the resulting trajectory data, i.e. body position and orientation over time and targets two goals. First, we would like to understand the general nature of the dance motion, i.e. what are frame properties like maximum velocities, angular precision and the size of the dance area. These global parameters define the target properties of the robot's mechanics and actuators. Secondly, we would like to learn from the observations which motion features are invariant throughout a large set of dances and are therefor likely to be of significance in the communication process. These key motion features are then implemented in the honeybee robot in order to generate realistic waggle dances.

We present a comprehensive list of dance characteristics, discuss interesting aspects of natural dances and show the trajectories generated by the final robotic dance model. This analysis gives a thorough description of the variability of the body motion of waggle dancers and thus might serve as the base of future analyses of the dance. The video recordings, the trajectory data, the matlab code for the model and all results of this analysis will be made available to the scientific community through a video and metadata management system on www.beetube.eu ([16]).

## Materials and Methods

### 2.1 Tracking the videos and resulting trajectories

**Figure 1:**
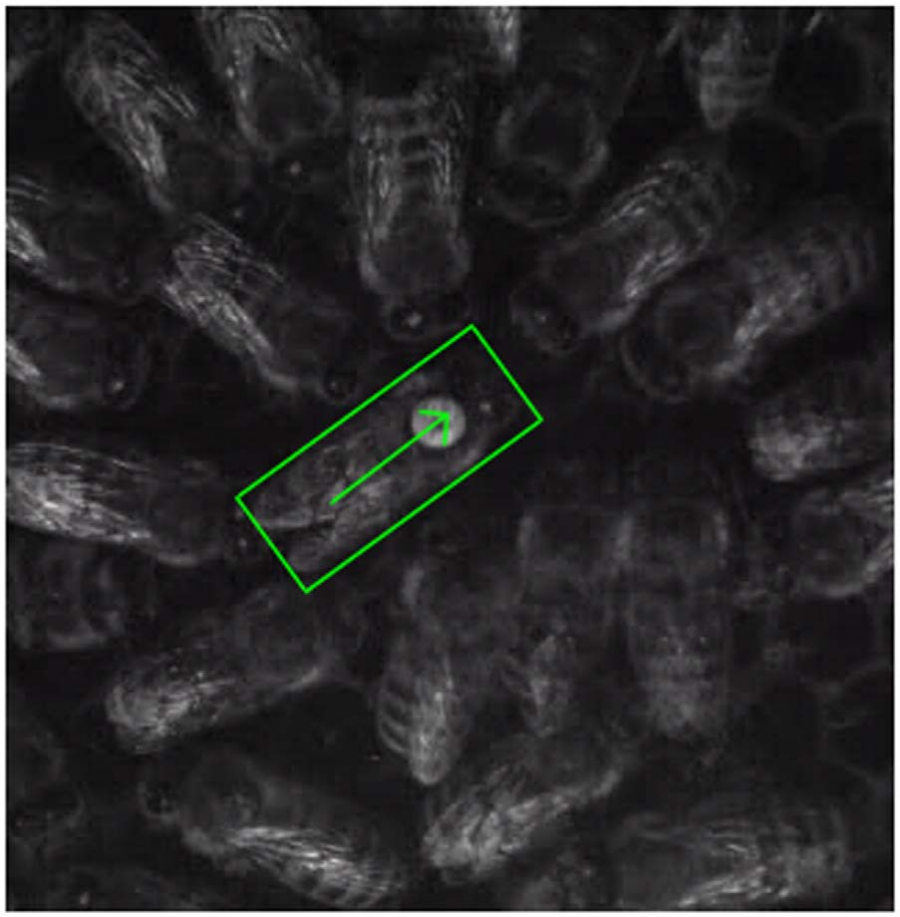
A frame of a highspeed video recording that was processed by the tracking program. The position of the dancer can be marked with a rectangular box by the user for a starting frame. The position of its center and the angle of the box are found and stored for consecutive frames automatically.

Video recordings (100 fps, VGA resolution) of waggle dances of European Honeybees (*Apis mellifera carnica*) were taken using a Basler A602f grayscale camera. We lit the comb surface with an array of red light LEDs (peak wavelength at 670 nm). Only recordings of dances advertising the same (230 m distant) feeder were used for the present analysis. The videos were then subjected to a tracking program [17] framewise. The program computed an automatic position that was checked by a person and corrected if necessary to produce reliable data. For every video frame the software stored the orientation and planar position of a bounding box (see Fig. 1) to a file. Because our video recordings have different magnification levels we measured the number of pixels of a unit distance (5 mm, a cell diameter) using a custom program. The trajectories were then translated to millimeter scale and rotated by the sun's azimuth angle and the feeder's direction in the field. After that rotation all dances were normalized,i.e. all waggles “point” to 0. Thus, all angular measures of dances of different day times were made comparable. The total number of waggle runs entering the analysis is 1009. They were obtained from 108 dances of 20 individuals.

### 2.2 Statistical Analysis of the Trajectories

Any tracked dance motion might be divided into a (hidden) dance model component and secondary, non-dance behaviors (e.g. evasive maneuvers, trophallaxis). We assume that the observed dance motion is a mixture of the dance motion and other motion that is superimposed or interspersed depending on external and internal stimuli. Thus, for the robotic dance it makes sense to make use of a model rather than playing back previously tracked dances. By analyzing many dance trajectories we expect that the influence of non-dance behaviour will cancel out statistically. To assess a minimal set of parameters with which to model a realistic dance we first made a list of numerous candidate properties of the trajectory data. We then discarded parameters showing high variance. Some of the remaining parameters were redundant. The parameter with lower variance was selected for the dance model. The trajectory data allows a vast variety of statistical queries. To retain order, we divide the parameters in sections “Global Dance Parameters”, “Waggle Parameters”, “Return Parameters” and “Intra-Waggle Parameters”. Table 1 enumerates the most important properties we extracted and classifies them into the forementioned categories. A complete list can be found in Appendix S1.

**Table 1:** Statistics of dance properties.

Parameter	Unit/Ann.	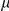	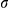	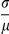
Global Parameters				
dance duration	s	5.24	0.85	0.16
dance radius	mm	-		
	x	-	7.6	-
	y	-	7.1	-
waggle-return	-	0.22	0.10	0.44
duration ratio				
Waggle Parameters				
duration	s	0.44	0.16	0.36
waggle length	mm	6.32	2.36	0.37
waggle orientation		−0.03	28.06	-
waggle direction		1.24	24.85	-
waggle drift	mm			
	forward	0.27	4.57	-
	sideward	−0.01	4.62	-
divergence A				
	orientation	31.98	-	-
	direction	22.11	-	-
divergence B				
	orientation	33.12	19	0.57
	direction	22.76	29.86	1.31
Return Parameters				
return duration	s	2.13	0.47	0.22
return velocity	mm s 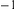	20.10	4.27	0.21
Intra-Waggle Parameters				
orientation		13.89	8.33	0.60
amplitude				
displacement	mm	2.64	1	0.38
amplitude				
waggle frequency	Hz	12.67	1.89	0.15
waggle velocity	mm s 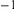	15.04	5.00	0.33
waggle steps	mm			
	forward	1	0.9	-
	sideward	0.04	0.9	-

An explanation of the parameters is given in the section 2.2. Units and annotations are shown in column 2. Means, standard deviation and coefficient of variation are given, if available.

#### 2.2.1 Global Parameters

**Figure 2:**
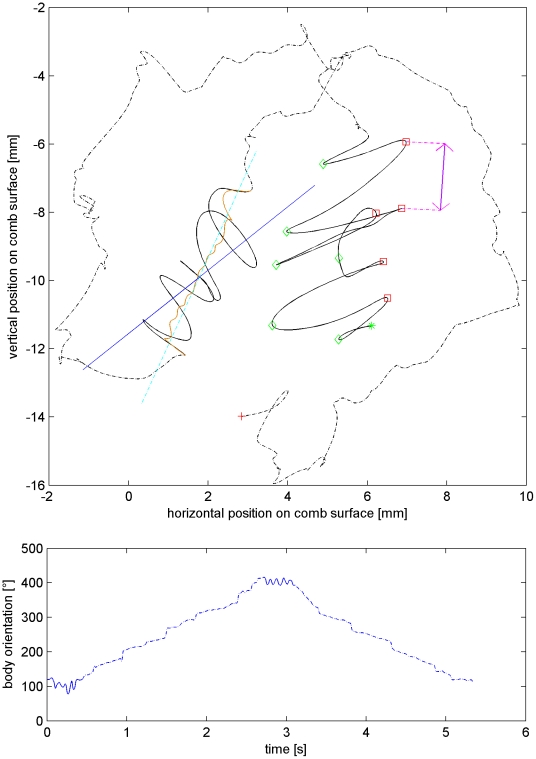
A natural dance period. Top: Plot of planar positions of the dancer bee's center. Two consecutive waggle runs are depicted. Return runs are dash-dotted. Start and end are marked with an asterisk and plus sign, respectively. On the left waggle run we show the linear least squares fit (dashed line) of the smoothed waggle (light grey solid curve) and the mean orientation line (solid line). In the right waggle run we marked the left and right turning points of the waggle oscillation with a diamond and square sign, respectively. Bottom: Orientation of the bounding box over time. Return run orientation is dash-dotted.

This section encloses parameters specific to a dance period, i.e. the sequence waggle-return-waggle-return (see Figure 2). These are: *dance duration* (the duration of a period in seconds), *dance area* (the spatial distribution of a dance period in millimeters), *dance orientation* (the mean angle of the dancer throughout a dance period) and *waggle-return duration ratio* (the ratio of consecutive waggle and return durations).

#### 2.2.2 Waggle Run Parameters

The waggle run class is specific to parameters describing a waggle run or relations between waggle runs. These are: *waggle duration* (the duration of a waggle run in seconds), *waggle length* (the length of a line connecting start and end point of a waggle run in millimeters), *waggle orientation* (the mean angle of all stored positions throughout the waggle run in degrees), *waggle direction* (the angle of a linear least-squares fit of the smoothed waggle run's planar positions in degrees), *waggle drift* (the displacement vector of two consecutive waggle runs) and *waggle divergence* (method A: the angle between the mean orientation (or direction) of all left and all right waggle runs; method B: the mean angle between consecutive waggles).

#### 2.2.3 Return Run Parameters

In this section parameters describing return run properties are clustered: *return run duration* (the duration of a return run in seconds), *return run forward velocity*, *return run sideward velocity* and *return run angular velocity* (the motion velocities in the return run as a function of time).

#### 2.2.4 Intra-Waggle Parameters

All parameters that describe intrinsic properties of waggle runs are specified in this class: The *orientation amplitude* (the orientation difference of the body in facing, consecutive turning points of a waggle oscillation), *displacement amplitude* (the distance of an turning point to the midpoint of the previous and following turning point), *waggle frequency*, *waggle velocity* (the forward velocity while waggling), *waggle steps* (the displacement vector of all left and right turning points).

A number of parameters were previously identified to be highly correlated to particular properties of the feeding location.

E.g. the angles *waggle orientation* and *waggle direction* correlate with the direction to the feeder. The *waggle length* or the *waggle duration*, the *return length* and *return duration* or even the number of waggle oscillations were described to correlate with the distance to the feeder ([1]). Our analysis also gives a numerical reason for the choice of one of these redundant parameters over the other. We assume that a lower variance indicates the conservation of that property and thus a high significance in the decoding process. After the parameter selection the model is supposed to create a dance that meets the frame properties that are described by the global parameters - these are not included explicitly in the model. They form the spatial and temporal limits of the robotic dance and serve only to verify the model.

Besides the properties known from the literature we describe the *waggle drift*, i.e. the vector connecting two consecutive waggle run's starting points. To decide whether to include the property into the list of dance parameters we test the hypothesis of different means with a Hotelling T-square test. We introduce the parameter *waggle step* as the vector connecting two consecutive left or right turning points of waggle oscillations. This might be of interest, because the turning points are likely to be the antennal “sampling points” of the waggle motion from the perspective of the dance followers. Also we introduce two measures for describing the amplitude of the waggle motion: *orientation amplitude* and *displacement amplitude*. The former is defined as the orientation difference at two consecutive turning points, the latter is defined as the distance of an turning point to the midpoint of the line that connects the one before and after.

The program MATLAB® is used for the whole analysis. We manually reviewed all automatically obtained results. First we run a script that identifies the waggle runs to obtain the separation needed for the class-specific analysis. Therefor we Fourier transform the one-dimensional derivatives of the x and y coordinates using a sliding window of 0.2 seconds width. A Fourier transform decomposes a signal into its constituent frequencies. If we find a spectral activity of the body motion at 12 Hz higher than a threshold the respective window was selected to contain tail-wagging. The resulting binary data was post-processed using dilution and erosion operations, known from image processing, to close gaps in some waggle runs. A few angular measures in the waggle phase were computed using two different methods. The “orientation method” is the angular mean of all stored orientations of the bounding box throughout the waggle phase. The “direction method” is the angle of the linear least squares fit of the 2D trajectory of a waggle run. The x,y positions were smoothed before the fit. This was necessary for very short waggle phases because these were “shorter than wide”, i.e. the fit would express the lateral motion rather than the forward motion. Smoothing the trajectory helps to extract the direction of the waggle, but might be a source of additional error and will be discussed later. Some authors have used one or the other method to measure the waggle angle ([18],[19],[20],[1],[2]). Interestingly, in some dances these angles differ significantly. I.e. some bees “look” into a direction different to the direction of their waggle path. Another program identifies the turning points of the waggle runs by finding the points on the trajectory that locally maximize the orthogonal distance to the mean direction line, as depicted in Figure 2. Some waggle runs were too short to be found by the automatic procedure since they consist of just one oscillation. We consider only waggle runs longer than two oscillations in the manual selection or 100 ms in the automatic one. Some return runs are interrupted, i.e. the subsequent waggle run is performed after a long time, after trophallaxis or other behaviors. These runs are not considered a return run and the waggle run that follows is not paired with the one before for the computation of the *waggle divergence*. The threshold for exclusion is 4 seconds (mean return run duration plus two standard deviations is around 3 seconds).

To compare the variability of parameters of different scales and units we compute the coefficient of variation (CoV) which is defined as the standard deviation divided by the mean. The two-dimensional test of difference of mean is performed using Hotelling's T-square statistic. Difference of means of one-dimensional data is done with a Student's t-test. Test for uniformity of circular data is performed with Rao's spacing test.

## Results

### 3.1 Global Parameters

The CoV of the *dance period duration* is 0.16. That is a hint to a very tight temporal dance regime. The standard deviation of the *dance period area* is around 7 mm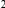. That means almost all dance periods are performed within an area of 28 mm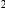, i.e. a square with edges two bee lengths long. The distribution of the *dance angle* is uniform (P = 0.5). That renders it likely that follower bees differentiate between the body pose of the dancer in waggle - and return runs. The CoV of the *waggle-return-ratio* is 0.44. Again, this points to the high importance of the temporal dynamics.

### 3.2 Waggle Run Parameters

The *waggle run duration* has low variance (CoV = 0.36). The means of the two angular measures *waggle direction* and *waggle orientation* are not significantly different (two tailed t-test, P = 0.3078). The mean drift vector was tested to be not significantly different to a zero drift (Hotelling T-square, F-Test, P = 0.178) meaning that dancing bees are not systematically progressing in a certain direction from waggle to waggle. The analysis of the *waggle divergence* reveals a difference in the orientation and direction measure. Showing similar variance, the difference of both divergence measures is extremely significant (P
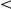
0.0001). The actual waggle path a dancer describes therefor gives a better approximation of the “real” direction to the feeder than the body orientation.

### 3.3 Return Run Parameters

**Figure 3:**
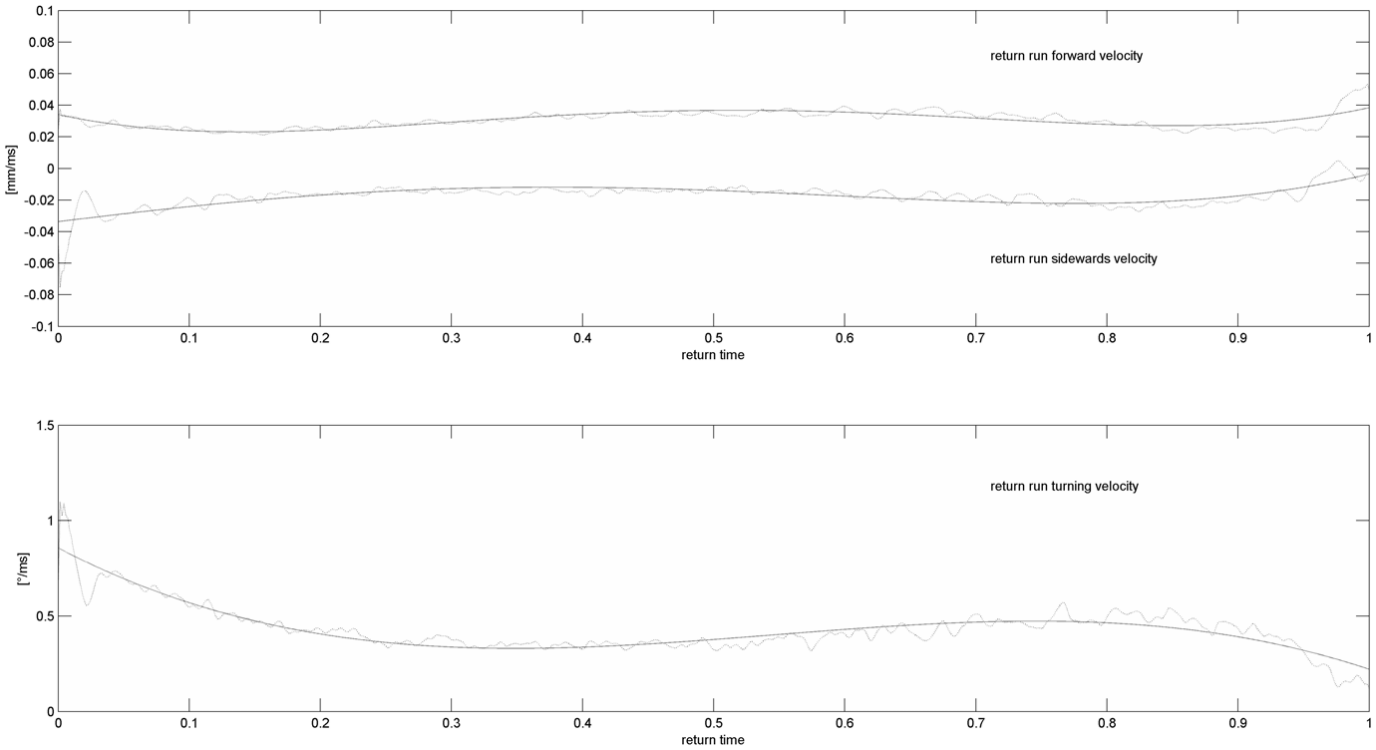
The forward, sideward and angular velocities, taken from 358 left and 362 right return runs and resampled to a unit duration (0 to 1). The average velocities are drawn dash-dotted, the solid line depicts the polynomial fit. The top plot shows the forward (top) and sideward (bottom) velocities, the second plot depicts the angular velocities.

The *return duration* and *return velocity* have a CoV of 0.22 and 0.21, respectively. We resampled forward, sideward and angular velocities in the return runs to vectors of unit length. By default return runs were downsampled to 700 elements - one third of the mean number of points. Afterwards, the vectors could be averaged and fitted using a polynomial model (see Figure 3). Both the forward and the sideward motion (for both left and right return phases) show interesting characteristics in the course of the return. A dancer turns fastest shortly after the waggle and slowest just before the following one. The amount of sideward motion is not negligible and always directed outwards.

### 3.4 Intra-Waggle Run Parameters

**Figure 4:**
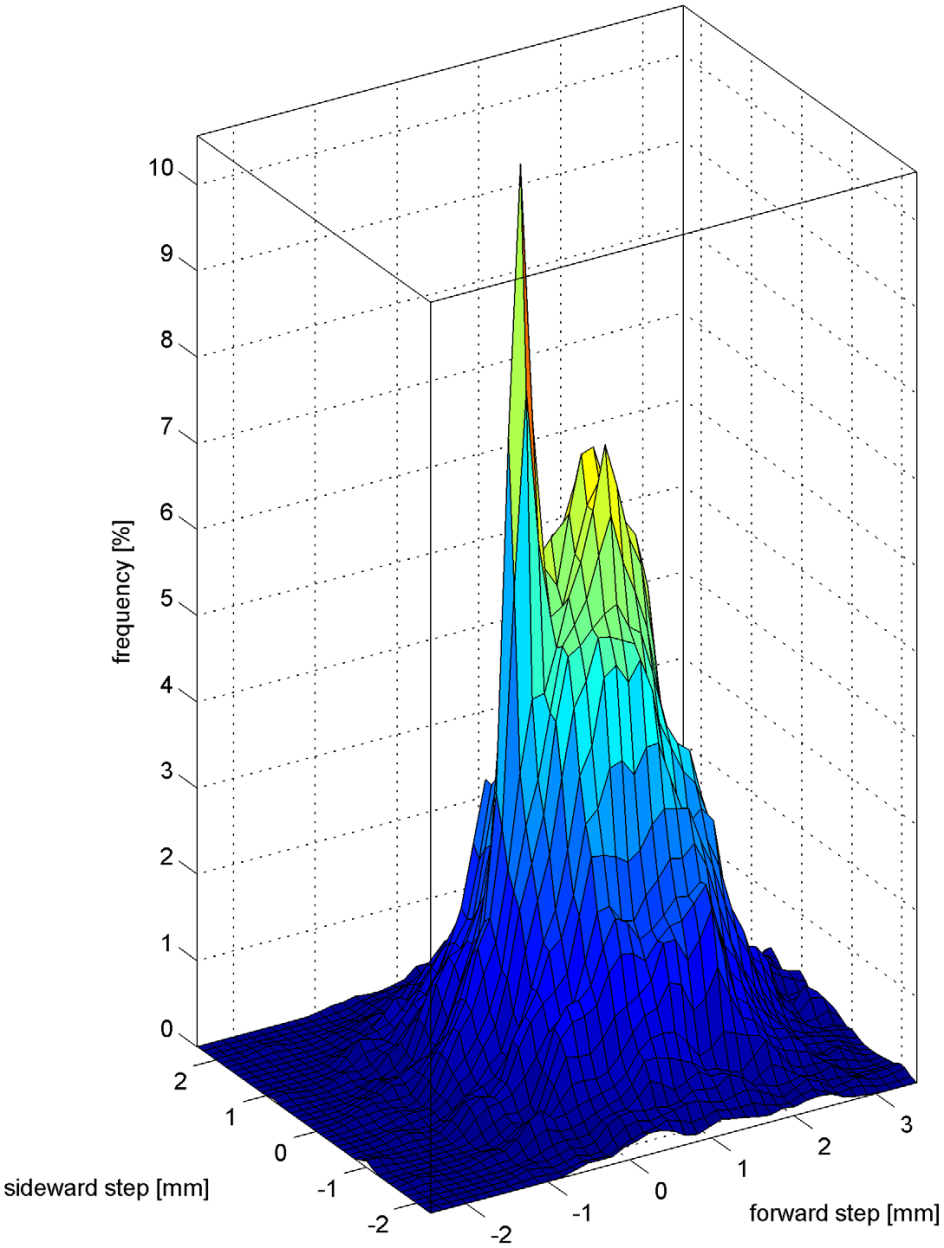
The histogram of the waggle step vectors exhibits two modes: a sharp peak at (0 mm, 0 mm) and another local maximum at 1 mm in the waggle direction. The former corresponds to wagging on the spot, the latter reflects the forward motion through one waggle movement.

The *orientation amplitude* is found to be more variable than the *displacement amplitude*. Coefficients of variation are 0.6 and 0.38, respectively. The *waggle frequency* band is rather narrow (CoV = 0.15). Since dances of 20 different dancers were pooled, one might expect the individual variance to be even lower. The speed of forward motion in the waggle phase also has low variance (CoV = 0.33). The *waggles steps* clearly point forward. A closer look at the distribution (Figure 4) reveals two modes: One peak at (0 mm,0 mm), i.e. a lateral waggle motion that has no forward movement and one peak at (1 mm,0 mm) that corresponds to the forward motion of the dancer after one single waggle oscillation. This sharp peak displays the steps a dancer makes in the waggle phase. Interestingly, the distribution has almost unit variance in both dimensions, i.e. the system shows comparable variability for the lateral wagging motion as for the forward motion.

### 3.5 The Robotic Dance Model

**Figure 5:**
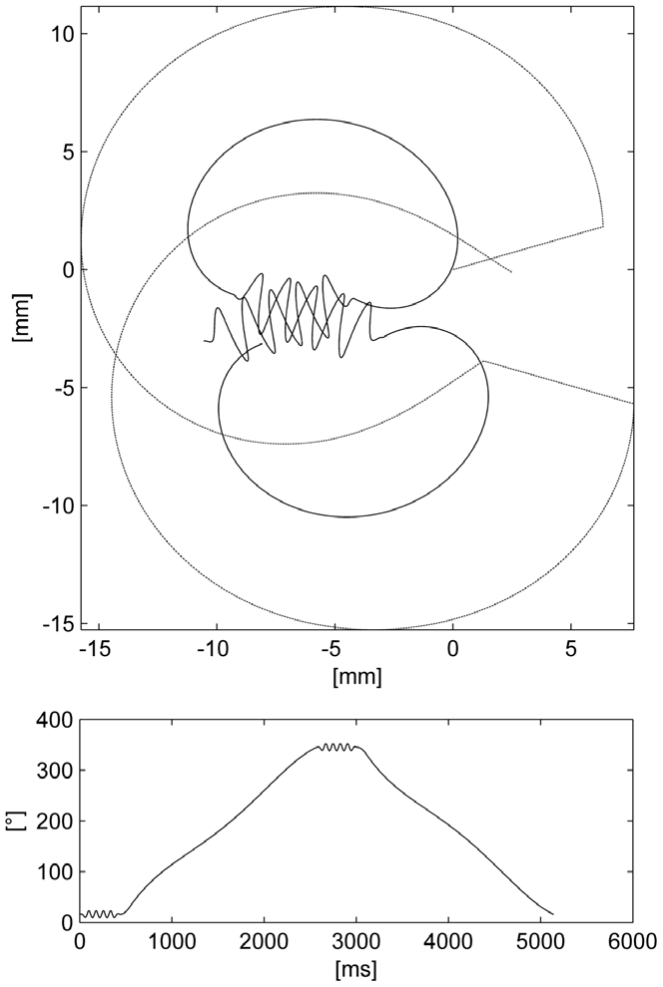
Top: A waggle dance generated from the default parameters. The solid line shows the trajectory of the center of the robot's body. The dash-dotted line depicts the curve of the 2D plotter that carries the motor used for the rotation and the lateral wagging motion. Bottom: Body orientation over time.

We include the following parameters in the dance model: *waggle duration*, *waggle forward velocity*, *waggle orientation*, *divergence*, *return forward velocity function*, *return sideward velocity function*, *return angular velocity function*, *displacement amplitude* and *waggle frequency*. Using these parameters a dance model was build and tested on our robot, a customized plotter ([14]). Since a waggle motion contains oscillations of around 13 Hz in all three dimensions (planar position and orientation) the robotic motion generated a lot of unwanted vibrations. To reduce the amount of mechanical noise we now only use the orientation motor for the waggle oscillation. The robot's body is excentrically fixed on the robot's arm such that the rotation axis of the robot points 1 cm in front of the robot body's head. Using this trick we can generate x,y-oscillations using only one motor. To reduce mechanical noise in the transition from return to waggle phase the waggle oscillation is multiplied with a linear ramp. Figure 5 shows a trajectory produced by the final model, which can be found as Matlab code in Appendix S1.

## Discussion

We propose dance parameters for the construction of a waggle dance model which produces trajectories closely resembling real ones. In this study we limited our analysis to obtain the parameters for the description of honeybee waggle dances advertising a fixed distance of 230 m. Future work will include recording, tracking and analyzing dances for a discrete set of distances.

The proposed low variance parameters indicate the significance of particular body pose or motion properties in the communication process. The way how the dancer's body moves and how this motion modulates other dance-related stimuli can, however, plausibly be allocated to and modeled with these particular properties. Furthermore, our evaluation allows some interesting inferences.

The mean angle of all positions throughout the dance (the *dance angle*) is uniformly distributed. No meaningful direction information can be obtained by sampling the body pose of the dancer throughout the whole dance. The waggle run, as the medium for the polar coordinates, needs to be highlighted in some way. It remains unknown if the waggle motion or other signals only present in the waggle phase, such as signals arising from wing oscillations, implement this. Both angular measures of the waggle exhibit a high variance. Averaging over all angles, however, yields a very good approximation of the direction to the feeder. The integration of subsequent samples of the waggle angle thus improves the precision of the message. De Marco et al. carried out a random resampling analysis to assess the error of the dance message as a function of the number of waggle runs sampled ([20]). Although random sampling might not be a realistic simulation, it underlines the great amount of uncertainty followers have to cope with when sampling a rather low number of waggle runs. The precision of the dance message might be increased by other signals though. The wing buzzes for example might mark the very body orientation that corresponds to the feeder's direction. Unfortunately, we were not able to detect the timing of the wing buzzes in our video recordings since the low lighting conditions we used did not allow for high shutter speeds and thus lead to a high amount of motion blur. In the coming season 2011 we will make new recordings in order to examine that hypothesis.

We found that the two angular measures yield a significant difference in the divergence angle. This angle is either assessed by comparing the means of left and right waggle runs (method A). Or it is measured “sequentially”, i.e. only consecutive waggle runs are used to collect the angular differences which, in the end, are averaged (method B). Both divergences differ with respect to the angular measure used. The orientation measure (based on the average body orientation) always yields larger divergences than the direction measure (that refers to the direction of the waggle path). This might be explained by the transitions of the waggle to return run or vice versa. While waggling, the dancer bee often turns her body into the return run's direction but keeps the body's trajectory straight. To this might add that at the beginning of the waggle the dancer bee might have turned not entirely into the right angle. To prove these assumptions we recalculated the divergences discarding the first and last 10%, 20% and 30% of the waggle run. Leaving out the first portions, the difference of the two divergences gets even larger, entirely on the account of the direction measure. By discarding the end of the waggle the difference of the two angular measures drops to 4

–5, which still is extremely significant (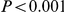, see Appendix S1 for the results of the recalculation). By discarding the first portion of the waggle we can not see an effect on the waggle orientation. That matches the fact that the angular and sideward velocities drop to almost zero right before the waggle run (see Figure 3). These results indicate that bees might finetune the divergence (tuned-error-hypothesis, see [21]).

**Figure 6:**
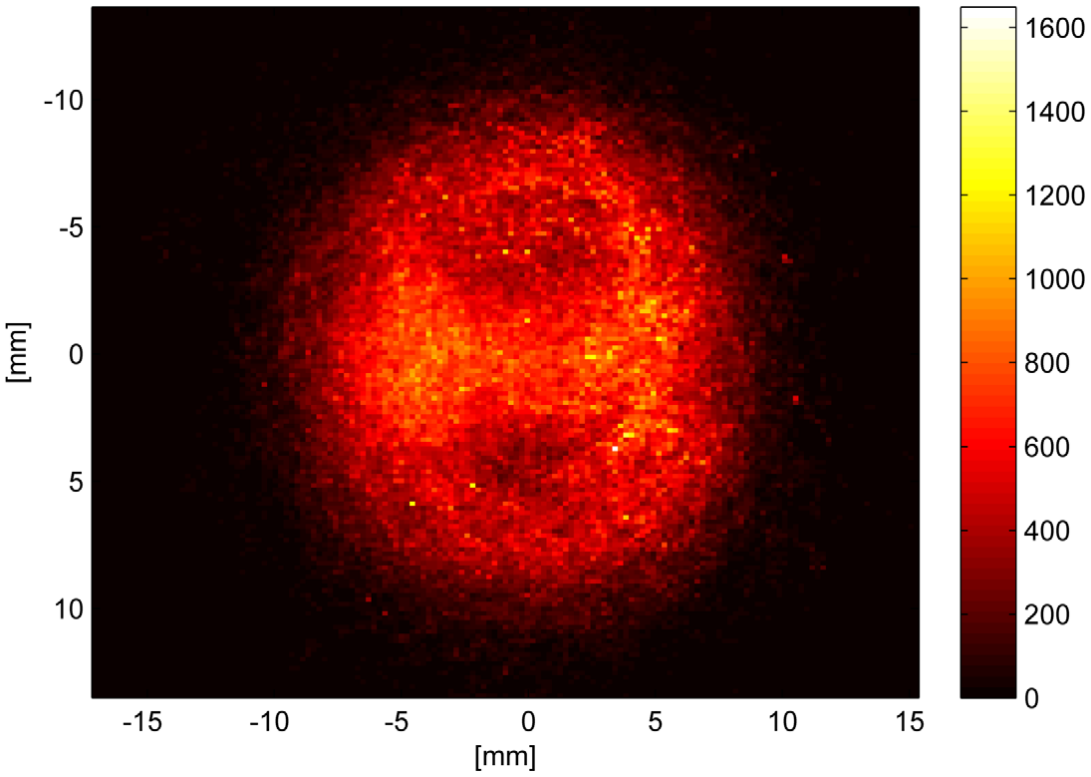
2D-Histogram of the body positions of dancing bees throughout 742 waggle periods (waggle-return-waggle-return). Dark regions denote a high frequency of presence. The trajectories were centered period-wise, i.e. we subtracted the mean from each of the W-R-W-R-paths.

Looking at real dance trajectories, the figure eight that is used commonly to describe the dance shape is observed rather infrequently. This is on the one hand due to the followers vigorous physical efforts to keep close body contact to the dancer and, by doing so, being obstacles in her way. On the other hand the dance floor is usually no free space and also the followers can not move freely. If the dancer's path is occupied she continues the turn on the spot or executes evasive maneuvers. Yet very effectively, a group of 2–3 followers usually clears the area for the dancer's subsequent waggle run with every turn they follow. We superimposed waggle periods (waggle-return-waggle-return) and created a two dimensional histogram of the body coordinates (Fig. 6). That makes visible the figure of eight and illustrates that a dance period takes place on a very small area of approximately 2 cm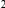. Together with the previous observation we infer that the return runs not only serve to return to the place where the previous waggle occured. It binds a distinctive group to the dancer and utilizes their motion to keep clear a yet small area within a very chaotic and dynamic environment. Also the tight temporal dynamics of waggle and return phase leads to the notion of a dance period as an “information packet”.

Applying Peirce's definition, communication codes can be of three kinds: iconic, indexical and symbolic ([22]). While iconic information relies on the resemblance of code and entity being communicated (first order code) and indexical codes are “pointing” to the object (second order code), symbols rely on the convention of a meaning. Potentially, the dance contains indexical (e.g. the smell of the crop) and iconic codes (the wing buzzes resemble the flight to be taken). Wether some dance components serve as symbols can not be answered based on the knowledge obtained from the observations and analyses we made only. Obviously, honeybees share a common brain function that maps the experience of the field trip and the hive situation to the very dance motion, or vice versa, that might as well be used to translate the multisensory input of a dance follower into information that is used to find the feeding spot. That common mapping function might be seen as a genetic convention and thus adds a symbolic character to the dance. Yet of now, we do not know how this function works in detail, wether it extracts such abstract concepts as angles and distances or maps the memory of a foraging experience to a motion pattern in a kind of “memory playback”. The honeybee robot alone might not enable us to reveal these internal processes completely. It can be of great help, though, answering which stimuli carry information. Using the robot we can decouple the stimuli involved and in doing so perform unnatural dances to observe the system under these conditions. Naturally, the degree to which the dance can be decomposed and distorted with the robot is generally unlimited. On the other side, the degree of resemblance of the imitation is bounded. This analysis yields a solid basis to model the dance motion with highly realistic results. It therewith lays the foundation to imitate the complex spatio-temporal dynamics of the associated stimuli. The proposed model is able to produce trajectories for a feasible, i.e. naturally observable, range of parameter values. We ran the model with natural parameter combinations (by measuring durations, motion speeds, etc. from video recordings) for dances advertising food sources at 300 m and 600 m distance and always obtain almost symmetric, figure-of-eight trajectory shapes. If we feed the model unnatural parameter sets, e.g. a high waggle run duration but a low return run duration, we would still obtain a trajectory - but only poorly resembling natural dance shapes. Furthermore, the model does not explicitely verify if the trajectory is resulting in motor speeds that exceed their limits. For a wide range of still plausible parameter sets however the robot is able to drive the path computed by the model.

So far, the robotic dance motion alone has not yet recruited followers to an unknown feeder. In a yet unpublished experiment, adding wing beats to the pure dance motion increases the rate with which foragers visit a known but depleted, unscented feeding site. Until now, we haven't yet been able to prove that the robot is communicating the direction to a feeder. This is closely related to the fact that followers have to sample a high number of waggle runs in order to gain a feasible estimate of the direction ([23]). In robotic dances a natural following behaviour is excited only partially. There might be three reasons for that observation. First, the implemented robotic stimuli are not a full replication of the natural stimuli. This includes a variety of aspects as the timing (e.g. of the wing buzzes), intensity (e.g. of the heat production) or spatial distribution (e.g. of the 3-dimensional field of air flows) of stimuli. Second, there might be some stimuli completely missing as well, e.g. substrate borne vibrations. Third, we have to consider the possibility that, although the robotic replication of the dance is sufficient, there might be other factors inhibiting the followers to actively execute their typical behavioral pattern. Disturbances may result from opening the hive, the unnaturally high forces when running over bees or aversive reactions to chemicals on the robot. Indeed, natural interactions on the comb surface are manifold and the acceptance of the robot seems to be a very fragile process. In order to reduce severe physical interferences, the robot must be stopped before running over the other bees. Manual control is not feasible with respect to precision and reaction time. Therefore we utilize small embedded camera modules that monitor a perimeter around the robot to recognize obstacles and trigger trophallaxis automatically ([14]). Also we have added a transparent plastic plate to the robot's central rod to cover the aperture of the hive through which we insert the robot. Future recordings of dances and upcoming recruitment experiments studying the ability of the robot to convey directional or distance-related information will provide the basis for the generalization of our current waggle dance model.

## Supporting Information

Appendix S1 This document contains calculations of different divergence measures, the Matlab code for the dance model and figures showing the distributions of most of the dance properties.
(PDF)
