# Supplementary material for: Analysis of the Waggle Dance Motion of Honeybees for the Design of a Biomimetic Honeybee Robot
Source: PLoS One. 2011 Aug 3;6(8):e21354. doi: 10.1371/journal.pone.0021354 (PMC3153927; doi:10.1371/journal.pone.0021354)
Supplement: Appendix S1 — This document contains calculations of different divergence measures, the Matlab code for the dance model and figures showing the distributions of most of the dance properties. (PDF) [file pone.0021354.s001.pdf]

Table 1: Recalculation of the divergence, i.e. the difference of left and right waggle angle. A portion of the beginning and end of the waggle trajectory was left out for the calculation of the waggle run orientation (average orientation) and the waggle run direction (average angle of path). This table shows the difference of these two measures obtained by substracting the means of all left and all right waggle run orientations / directions (method A). Originally, the difference was 9.87 ° (31.98 ° vs. 22.11 °)

Portion Removed	Difference	Orientation vs. Direction
Beginning		
10%	10.80 °	31.70 ° vs. 20.90 °
20%	13.66 °	31.47 ° vs. 18.08 °
30%	14.54 °	32.87 ° vs. 17.73 °
End		
10%	7.57 °	31.04 ° vs. 22.35 °
20%	4.18 °	29.72 ° vs. 24.44 °
30%	4.14 °	29.40 ° vs. 24.13 °

Table 2: Recalculation of the divergence using method B (mean difference of consecutive waggle runs). Original difference: 10.36 ° (33.1 ° vs. 22.76 °)

Portion Removed	Difference	Orientation vs. Direction
Beginning		
10%	11.38 °	32.80 ° vs. 21.45 °
20%	14.08 °	32.90 ° vs. 18.80 °
30%	15.08 °	33.40 ° vs. 17.86 °
End		
10%	8.15 °	31.04 ° vs. 22.89 °
20%	4.77 °	29.72 ° vs. 25.04 °
30%	5.33 °	29.40 ° vs. 24.07 °

Return Run: motion velocity function parameters Return velocity – General model:
Coefficients (with 95% confidence bounds):

$$curve\_speedx(x) = p0 * x^4 + p1 * x^3 + p2 * x^2 + p3 * x + p4$$

$p0 = 0.728(0.6792, 0.7768)$
$p1 = -1.466(-1.564, -1.367)$
$p2 = 0.9225(0.857, 0.988)$
$p3 = -0.1805(-0.1966, -0.1644)$
$p4 = 0.03409(0.03293, 0.03525)$

$$curve\_speedy(x) = p0 * x^4 + p1 * x^3 + p2 * x^2 + p3 * x + p4$$

$p0 = 0.2683(0.2002, 0.3364)$
$p1 = -0.2951(-0.4325, -0.1578)$
$p2 = -0.04566(-0.137, 0.04571)$
$p3 = 0.1027(0.08023, 0.1251)$
$p4 = -0.0337(-0.03532, -0.03209)$

Dance model: Matlab code

```
%neues Tanzmodell
function v = new_dance_model()
global waggle_angle waggle_divergence waggle_speed waggle_duration
global waggle_ramp_length waggle_freq waggle_amp_xy waggle_amp_a
global return_speed return_duration return_orientation_function
global return_velocityX_function return_velocityY_function dt T drift
global return_speed return_duration return_orientation_function dt T drift
global Qglobal pos exc return_speed_function_offset
global return_orientation_function_offset turn_scale return_orientation_function

%parameter
waggle_angle = 0; % 0   nach oben, ccw
waggle_divergence = 29.2667 * pi / 180; %default im paper: 32
waggle_divergence = 32 * pi / 180; %default im paper: 32
waggle_speed = 15.04; % in mm/s vortrieb des waggle runs
waggle_duration = 440; % in ms
waggle_freq = 12.67; % in Hz
waggle_amp_a = 6.9 * pi / 180; % in [0,2*pi] %hier peak to baseline angeben!!!
return_speed = 20.1; % in mm/s
return_duration = 2130; % in ms
exc  = 11;
waggle_ramp_length = 150; %[ms]

resamling_steps = 700;
resampling_coeff = resamling_steps / return_duration;

%return orientation velocity
%        return_orientation_velocity(x) = p1*x^3 + p2*x^2 + p3*x + p4
%      Coefficients (with 95% confidence bounds):
%        p1 =      -4.581  (-4.767, -4.395)
%        p2 =       7.517  (7.234, 7.801)
%        p3 =      -3.572  (-3.694, -3.45)
%        p4 =      0.8562  (0.8421, 0.8703)
p1 =      -4.581;
p2 =       7.517;
p3 =      -3.572;
p4 =       0.8562;
func_string = sprintf('%f*(%f*x.^3 + %f*x.^2 + %f*x + %f)', ...
                    resampling_coeff, p1, p2, p3, p4)
return_orientation_function = inline(func_string);
                    %t = linspace(0, 1, return_duration);
                    %plot(t, return_orientation_function(t))

%we have to scale the return_orientation_velocity by turn scale to be in
%the right pose when entering the next waggle
t = linspace(0,1,return_duration);
f = return_orientation_function(t);
turn_scale = (360-waggle_divergence*180/pi)/sum(f)

%return velocity
% curve_speedx =
%
%      General model:
%        curve_speedx(x) = p0*x^4 + p1*x^3 + p2*x^2 + p3*x + p4
%      Coefficients (with 95% confidence bounds):
%        p0 =       0.728  (0.6792, 0.7768)
%        p1 =      -1.466  (-1.564, -1.367)
%        p2 =      0.9225  (0.857, 0.988)
%        p3 =     -0.1805  (-0.1966, -0.1644)
%        p4 =     0.03409  (0.03293, 0.03525)
p0 =        0.728;
p1 =       -1.466;
p2 =        0.9225;
p3 =       -0.1805;
p4 =        0.03409;
func_string = sprintf('%f*(%f*x.^4 + %f*x.^3 + %f*x.^2 + %f*x + %f)', ...
                    resampling_coeff, p0, p1, p2, p3, p4);
```

```
return_velocityX_function = inline(func_string);


% curve_speedy =
%
%      General model:
%        curve_speedy(x) = p0*x^4 + p1*x^3 + p2*x^2 + p3*x + p4
%      Coefficients (with 95% confidence bounds):
%        p0 =      0.2683  (0.2002, 0.3364)
%        p1 =     -0.2951  (-0.4325, -0.1578)
%        p2 =    -0.04566  (-0.137, 0.04571)
%        p3 =      0.1027  (0.08023, 0.1251)
%        p4 =     -0.0337  (-0.03532, -0.03209)
p0 =        0.2683;
p1 =       -0.2951;
p2 =        -0.04566;
p3 =       0.1027;
p4 =        -0.0337;
func_string = sprintf('%f*(%f*x.^4 + %f*x.^3 + %f*x.^2 + %f*x + %f)',  ...
                    resampling_coeff, p0, p1, p2, p3, p4);
return_velocityY_function = inline(func_string);


close all

figure
plot(return_velocityX_function(t))
hold
plot(return_velocityY_function(t))

sum(return_velocityX_function(t))
sum(return_velocityY_function(t))

%velocities
v = [];
%initial position
pos = [0 0 waggle_divergence/2];
%?
Q = 0;

for i = 1:1
    %1st waggle
    for t = linspace(0, waggle_duration, waggle_duration)
        q = getWaggleVelocity(t,1);
        pos = pos + q; %cumulative sum
        v = [v; pos]; %positions
    end


    %1st return
    for t = linspace(0, return_duration, return_duration)
        q = getReturnVelocity(t, pos(3),1);
        pos = pos + q;
        v = [v; pos];
    end

    pos

    %2nd waggle
    for t = linspace(0, waggle_duration, waggle_duration)
        q = getWaggleVelocity(t,0);
        pos = pos + q; %cumulative sum
        v = [v; pos]; %positions
    end

    %2nd return
    for t = linspace(0, return_duration, return_duration)
        q = getReturnVelocity(t, pos(3),0);
        pos = pos + q;
        v = [v; pos];
```

```
    end
end


size(v)
figure
hold off
subplot(2,1,1)
plot(v(:,1), v(:,2), 'g-.');
hold on

v(:,1) = v(:,1) - exc*cos(v(:,3));
v(:,2) = v(:,2) - exc*sin(v(:,3));

plot(v(:,1), v(:,2), 'r');

axis image

subplot(2,1,2)
plot(v(:,3)*180/pi)

function v = getWaggleVelocity(t, left)
global waggle_angle waggle_divergence waggle_speed waggle_duration
global waggle_ramp_length waggle_freq waggle_amp_xy waggle_amp_a
global return_speed return_duration return_orientation_function dt T drift
%return value
v = [0 0 0];

%number of waggle periods
num_periods = ((waggle_duration/1000)*waggle_freq);
sample_points_for_one_period = 1000 / waggle_freq;

if (left)
    sign = 1;
else
    sign = -1;
end

ramp = 1;
num_periods = (waggle_duration/1000.0) * waggle_freq;
sample_points_for_one_period = 1000.0 / waggle_freq;

%damp one period at start and end
if (t < sample_points_for_one_period)
    ramp = t / sample_points_for_one_period;
else
    if (t > (sample_points_for_one_period * (num_periods - 1)))
        ramp = (waggle_duration - t) / sample_points_for_one_period;
    end
end

%+ 0.5 := sample the cosine symmetrically
%make it in [0,1]
%t_prcnt = ((t+.5)/waggle_duration);
t_prcnt = (t/waggle_duration);
num_periods = ((waggle_duration/1000)*waggle_freq);

%v(3) = waggle_amp_a * cos( ((waggle_duration / 1000)*waggle_freq) *
%t_prcnt * 2 * pi) / arc;

v(3) = waggle_amp_a * ramp * cos(num_periods * t_prcnt * 2 * pi) / ...
                         (1000 / (waggle_freq * 4) / (pi*0.5)) ;
v(1) = waggle_speed * cos(waggle_angle + sign*waggle_divergence/2) / 1000;
v(2) = waggle_speed * sin(waggle_angle + sign*waggle_divergence/2) / 1000;

function v = getReturnVelocity(t, Ocur, left)
global turn_scale return_duration exc return_orientation_function
global return_velocityX_function return_velocityY_function
v = [0 0 0];
if (left)
```

```
    sign = 1;
else
    sign = -1;
end

ramp = 1;
%damp one period at start and end
d = 100;
if (t < d)
    ramp = t / d;
end
% unit_t = (t+0.5) / return_duration;
unit_t = t / return_duration;

v(3) = turn_scale*sign*ramp*pi*return_orientation_function(unit_t)/180;
% v(1) = (return_velocity_function(unit_t) * cos(Ocur + v(3)) / 1000) ...
                        + exc*(cos(Ocur+v(3))-cos(Ocur));
% v(2) = (return_velocity_function(unit_t) * sin(Ocur + v(3)) / 1000) ...
                        + exc*(sin(Ocur+v(3))-sin(Ocur));
%%ACHTUNG!! x und y veocities zur ck drehen um
% Ocur !!!!
v_fwd = turn_scale*ramp*return_velocityX_function(unit_t);
v_swd = turn_scale*ramp*sign*return_velocityY_function(unit_t);
c = cos(Ocur);
s = sin(Ocur);
%rotate
v(1) = c*v_fwd - s*v_swd + exc*(cos(Ocur+v(3))-cos(Ocur));
v(2) = s*v_fwd + c*v_swd + exc*(sin(Ocur+v(3))-sin(Ocur));
```

Table 3: Dance Properties

Parameter	Unit / Ann.	μ	σ	$\frac{\sigma}{\mu}$
**Global Parameters**				
dance duration	s	5.24	0.85	0.16
dance periode area	mm			
	x	-	4.8	-
	y	-	5.0	-
dance radius	mm			
	x	-	7.6	-
	y	-	7.1	-
waggle-return duration ratio	-	0.22	0.10	0.44
dance orientation	°	80.6	56.6	-
**Waggle Parameters**				
duration	s	0.44	0.16	0.36
waggle length	mm	6.32	2.36	0.37
waggle orientation	°	-0.03	28.06	-
waggle direction	°	1.24	24.85	-
direction vs. orientation	-	-4.4	15.23	-3.46
waggle drift	mm			
	forward	0.27	4.57	-
	sideward	-0.01	4.62	-
divergence A	°			
	orientation	31.98	-	-
	direction	22.11	-	-
divergence B	°			
	orientation	33.12	19	0.57
	direction	22.76	29.86	1.31
**Return Parameters**				
return duration	s	2.13	0.47	0.22
return velocity	mm s^{-1}	20.10	4.27	0.21
return path length	mm	42.2	8.39	0.2
**Intra-Waggle Parameters**				
orientation amplitude	°	13.89	8.33	0.60
displacement amplitude	mm	2.64	1	0.38
waggle frequency	Hz	12.67	1.89	0.15
waggle velocity	mm s^{-1}	15.04	5.00	0.33
waggle steps	mm			
	forward	1	0.9	-
	sideward	0.04	0.9	-

This table represents a list of dance statistics. An explanation of the parameters is given in the section: Statistical Analysis of the Trajectories. Units and annotations are shown in column 2. Means, standard deviation and coefficient of variation are given, if available.

Figures

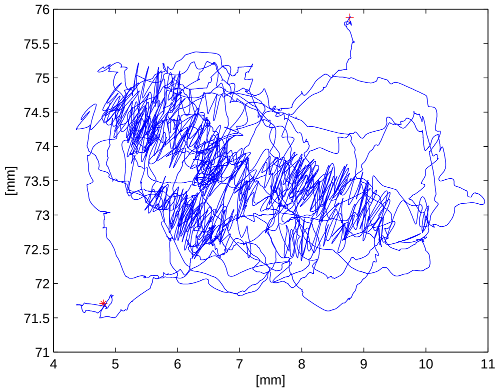

(a) A sample trajectory, i.e. body positions through time, of a full dance.

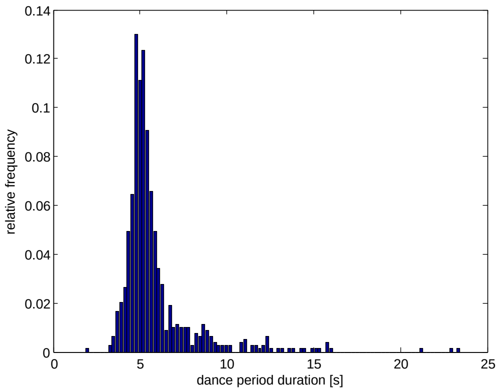

(b) Dance period duration histogram.

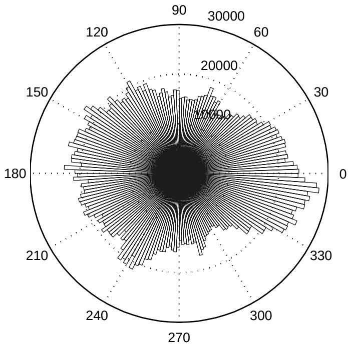

(c) Histogram of all orientations throughout a dance.

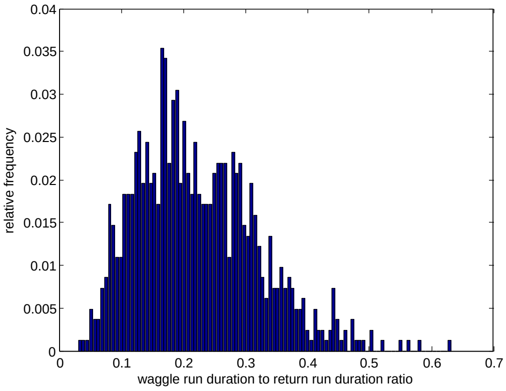

(d) Histogram of waggle-return duration ratios of consecutive waggle and return runs.

Figure 1: Global Parameters

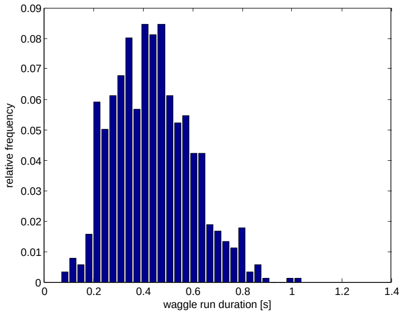

(a) Histogram of waggle run durations.

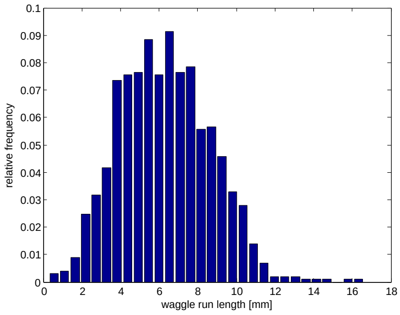

(b) Histogram of waggle run lengths.

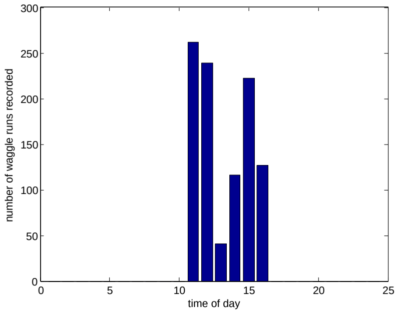

(c) Number of waggle runs sampled over day time

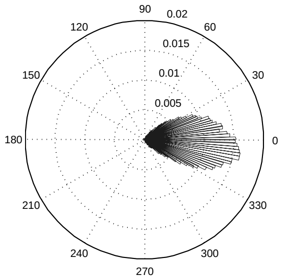

(d) Histogram of waggle orientations.

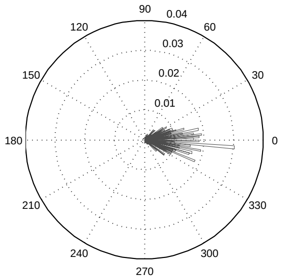

(e) Histogram of waggle directions.

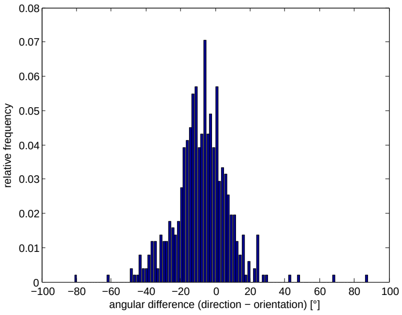

(f) Histogram of differences (direction - orientation)

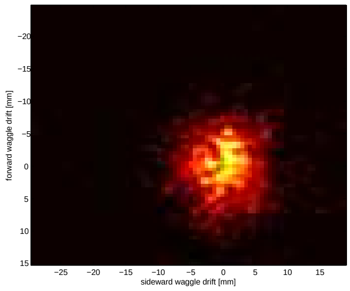

(g) Histogram of waggle drift vectors.

Figure 2: Waggle Parameters Part I

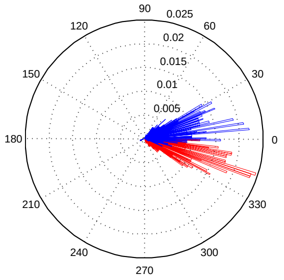

(a) Histogram of left (blue) and right (red) waggle run orientations

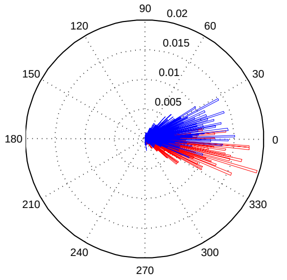

(b) Histogram of left (blue) and right (red) waggle run directions

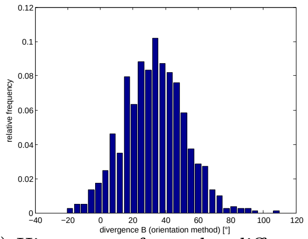

(c) Histogram of angular differences of consecutive waggle run orientations

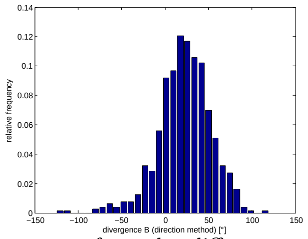

(d) Histogram of angular differences of consecutive waggle run directions

```
WAGGLE DIVERGENCE (DIFFERENCE OF MEANS):
497 left and 510 right waggles
    Orientation:
mean left    (14.62, std:19.24)
mean right   (−17.36, std:14.17)
divergence: 31.98
    Direction:
mean left    (12.55, std:24.94)
mean right   (−9.56, std:19.52)
direction:  22.11

WAGGLE DIVERGENCE (MEAN OF CONSECUTIVE DIFFERENCES):
804 waggle pairs
    Orientation:
mean:        :33.12
std:         :19.00
CoV:         :0.57
    Direction:
mean:        :22.76
std:         :29.86
CoV:         :1.31
```

Figure 3: Waggle Parameters Part II

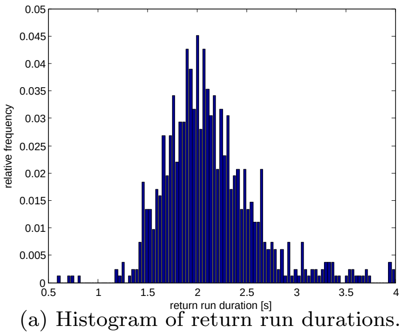

(a) Histogram of return run durations.

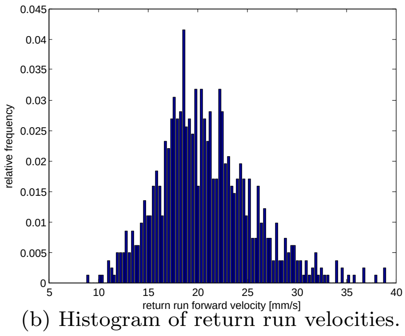

(b) Histogram of return run velocities.

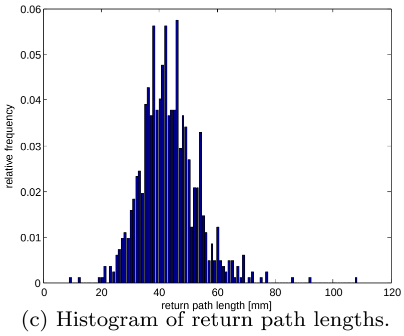

(c) Histogram of return path lengths.

Figure 4: Return Parameters

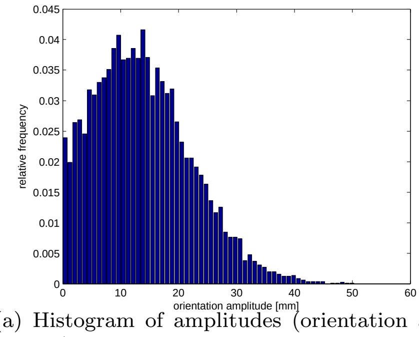

(a) Histogram of amplitudes (orientation amplitude).

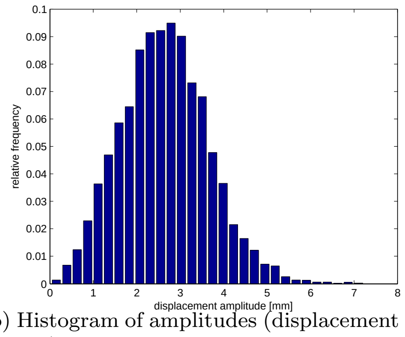

(b) Histogram of amplitudes (displacement amplitude).

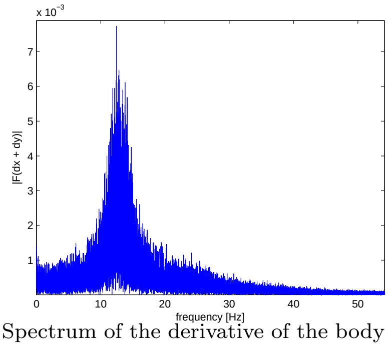

(c) Spectrum of the derivative of the body motion of the waggle run.

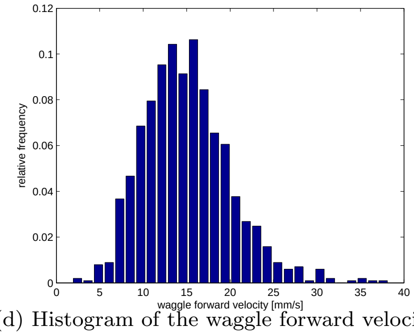

(d) Histogram of the waggle forward velocity.

Figure 5: Intra-Waggle Parameters Part I

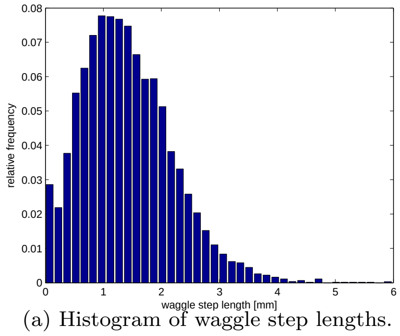

(a) Histogram of waggle step lengths.

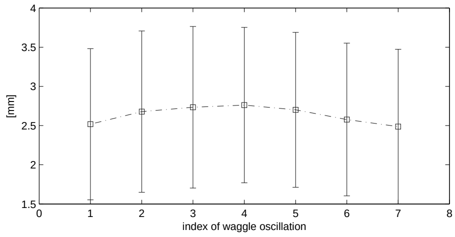

(b) Mean waggle amplitude for each oscillation.

Figure 6: Intra-Waggle Parameters Part II
